# Students Sense of Belonging and Academic Performance via Online PBL: A Case Study of a University in Hong Kong during Quarantine

**DOI:** 10.3390/ijerph19031495

**Published:** 2022-01-28

**Authors:** Xunqian Liu, Yi Yang, Jessica Ws Ho

**Affiliations:** 1 School of Humanities, Shanghai Jiao Tong University, Shanghai 200240, China; lxunqian@sjtu.edu.cn; 2 Chinese Language Centre of School of Chinese, The University of Hong Kong, Hong Kong 999077, China; u3002811@connect.hku.hk

**Keywords:** adapting to online teaching, problem-based learning, isolation

## Abstract

Innovative educational adaptations have been essential during the COVID-19 pandemic. Against the backdrop of school closures in Hong Kong resulting from unrest and COVID-19, this study proposed using problem-based learning (PBL) in online courses and empirically examined the influence of the PBL learning methodology on online learners’ sense of classroom belonging and academic performance. A total of 44 sophomores pursuing electrical and electronic engineering majors and taking “Practical Chinese for engineering students” as a compulsory course at the University of Hong Kong participated in the study. They were divided evenly between the experimental group C1 and the control group C2 (22 in each). We implemented online PBL learning for the experimental group, C1, and traditional online learning for the control group, C2. Quantitative data were collected via an achievement test and a scale on sense of classroom community. Qualitative data were obtained through a semi-structured focus group discussion. The quantitative results showed that the students who received the PBL learning method scored higher on tests, experienced a stronger sense of classroom belonging, and had closer connections with each other. A content analysis of student interviews revealed that implementation of the PBL learning method in online teaching could strengthen students’ classroom interactions and that the enhancement of their mutual connections could stimulate learning and improve learning efficiency. Overall, this study finds that the PBL learning method is effective in promoting students’ deep active learning and sense of community in the online environment.

## 1. Introduction

The 2019–2020 academic year was challenging for all universities in Hong Kong. In September 2019, the 2019 Anti-Extradition Bill movement triggered widespread unrest. Protesters scaled up their resistance by launching the “Dawn Operation” campaign that included constructing road obstructions, reinitiating the three suspensions (of work, classes, and markets) and other protest activities. The protest reached its climax during two fierce confrontations at the Chinese University of Hong Kong and the Hong Kong Polytechnic University. The conflict at Hong Kong Polytechnic University was particularly serious; one thousand people—mostly students—were trapped on campus for several days. In November 2019, campuses were occupied and the threat to student security led to the suspension of in-person classes. After resuming in January 2020, classes were again suspended in February due to the rapid spread of COVID-19, and higher education institutions shut down all their facilities. During the coronavirus pandemic, virtual learning was essential [1]. This forced universities across Hong Kong to transition to online teaching for the spring semester, so course instructors needed to adjust their courses to make them suitable for online learning.

Students’ sense of belonging refers to the experience of being cared for, respected, and valued by the campus community and other individuals on campus, such as faculty members and peers. It impacts students’ response and performance in academia. Studies have shown that distance education could develop some sense of belonging and integration through group tasks, shared documents, video conferences, email or even integrating social media into an online course [2]. In most online teaching environments, students rarely have direct contact with one another. Thus, online teaching needs to design more interactive activities to make students feel less isolated. Research has already revealed an alarming decline in students’ psychological health during the COVID-19 quarantine period, with some students suffering from anxiety and depression or succumbing to excessive drinking and other negative symptoms [3]. Social isolation can exacerbate the risk of distress and anxiety, thus contributing to poorer mental health [4]. Students in Hong Kong had to stay at home without attending school for a much longer period than students in other regions. In particular, with the escalation of protests in Hong Kong, news about protesters setting fires to blocks or attacking pedestrians kept surfacing, deepening students’ sense of helplessness and vulnerability. Thus, the question of how to help them concentrate on their studies and create feelings of connection and “togetherness” in online courses became increasingly urgent during quarantine.

Against this backdrop, this study examined a learning method that could facilitate students’ autonomous learning and encourage them to actively engage and cooperate with their peers. After in-depth discussions and analysis, we proposed a learning method that features problem-based learning for the online learning environment. Problem-based learning (PBL) is a widely recognized method for promoting student-centered learning, which contributes significantly to students’ problem-solving skills, self-directed learning skills, and team skills [5]. Meanwhile, PBL requires students to collaboratively learn in groups, thereby effectively enhancing mutual contact among themselves [6]. Self-exploration and self-directed learning methods can also strengthen students’ memorization of acquired knowledge [7]. Thus, aiming to serve as a reference for college-level distance learning programs during the COVID-19 quarantine period, we examined whether the collaborative learning-based online PBL learning method could strengthen students’ sense of classroom belonging and improve their performance.

The course used for this study, “Practical Chinese for Engineering Students”, is designed to instill a mastery of the varieties of the Chinese language as used in the field of engineering. As students in Hong Kong generally learn Chinese as their second or third language, the main objective of this course is to promote the professional use of the Chinese language in the field of engineering. It aims at helping students to master the skills of letter writing, email writing and target-oriented proposal writing in their profession. In the Chinese characters component, there are drilling practices to familiarize the students with the frequently used terms in their simplified forms. A key focus is on the use of Mandarin in technical negotiation.

## 2. Background Literature

### 2.1. PBL and Online Education

PBL is an integrated learning module that focuses on student-centered learning, problem-solving, collaborative learning, and group discussion [7]. PBL is well-known for its application in medical education, first noted at McMaster University in Canada in the 1960s [8]. The PBL approach to learning does not require students to have mastered a body of knowledge before they complete a project (as in typical undergraduate or master’s thesis), but instead allows students’ understanding and problem-solving abilities to evolve together. According to Hansen [9], the goals of PBL are to help students: (a) think critically, analyze, and solve complex real-world problems; (b) find, evaluate, and use learning resources; (c) work cooperatively in teams; (d) demonstrate effective communication skills; and (e) use content knowledge and intellectual skills to become continual learners.

Over the past decade or so, mixed teaching methods that combine PBL and online teaching have become increasingly popular. Erickson [10] found that online forums enable students to acquire feedback quickly from both tutors and lecturers. Compared with the traditional PBL learning method, the online PBL learning method allows students to be flexible in adjusting their learning schedules, so that they feel more motivated to learn. Students can study online anywhere they want and engage in discussions free from time and space limitations. Lajoie et al. [11] proposed that the relatively small PBL class size does not impose a high demand on network speed; thus, high quality synchronous online communication is feasible.

Many researchers have reported their online learning implementation of PBL during the COVID-19 situation. Foo’s study showed that the performance of students utilizing online PBL tutorials was lower than that of students participating in the conventional face-to-face approach [12]. Because online PBL tutorials might have appeared ‘unreal’ for learning and students need psychological adaptation. However, a study on clinical teaching, found the opposite. It reported that using online PBL was well-received by undergraduate students. Tutors and students could still maintain even better interaction due to the absence of such constraints as space and the reduction of possible anxiety associated with face-to-face discussion in traditional PBL [13]. Haslam et al. observed that students who have worked in PBL project groups had a more positive experience with online learning, which indicates that belonging to a project group can increase students’ motivation to participate in online teaching activities [14]. However, more studies are needed to analyze online PBL’s appropriateness, its application and practices, its strengths and weaknesses and its potential influence on different disciplines and at different levels.

### 2.2. Overcoming Isolation in Distance Learning

It is generally believed that classrooms are social environments in which social interactions with teachers and classmates shape students’ learning processes [15]. Therefore, classroom composition may play a crucial role in students’ motivational and learning outcomes. Classrooms can foster connections among students, enabling them to set their learning objectives, make friends, work together, and gain the satisfaction of learning in a classroom. Palloff and Pratt highlighted the relationship between the classroom and collaborative learning, claiming that collaborative learning is based in the classroom, and that the classroom can promote collaborative learning abilities [16].

Although many students enjoy the flexibility afforded by online courses, one disadvantage of online learning is that it exposes students to experiences of isolation and alienation. Indeed, students’ sense of isolation is an important challenge in online learning. Specifically, the sense of isolation in online educational settings can result in lower student persistence, lower student achievements, and higher stress levels [17]. These potential effects can undermine the quality of students’ online learning experiences.

PBL involves students examining problems in small groups and defining the additional knowledge and investigation required to solve the problems [10]. In many ways, PBL is as much about diagnosing the key unknowns in problems and identifying appropriate ways to tackle these problems as it is about solving the problems at hand. Therefore, PBL could serve as a means of facilitating collaborative learning and thus increasing interaction among students and addressing the decreased sense of belonging in online learning.

## 3. Theoretical Framework

### 3.1. Rationale for Using PBL for Practical Language Learning

Technical communication and technical writing skills are widely acknowledged to be critical for professionals in the STEM (Science, Technology, Engineering and Mathematics) fields [18]. Nowadays, engineering work has become more collaborative and horizontal. Thus, engineering students need to be able to effectively communicate in order to be fully prepared for future professional workplaces. Much has been written about the importance of getting engineering students to write [19,20] There is also study on language learning and writing development for specific purposes, focusing on pedagogical content for STEM students studying languages other than English at the university level [21]. However, little has been published on new teaching methods for STEM students’ language learning in higher education contexts. Given this gap, our research attempts to apply PBL to students’ practical language learning.

A large advantage of implementing PBL in language courses is that it encourages students to gain a deeper sense of understanding. Superficial learning is often a problem in language education, for example when students, instead of acquiring a sense of when and how to use which vocabulary, learn all the terminologies and expressions they will need for the exam and then promptly forget them. In a PBL classroom, this is combated by always introducing the vocabulary in a real-world situation, rather than as words on a list, and by activating the student; students are not passive receivers of knowledge but are instead required to actively acquire the knowledge.

### 3.2. Research Questions

A significant relation was found between the PBL method and collaborative learning, disciplinary subject learning, iterative learning and authentic learning, which, in turn, produced student engagement [7]. Based on the above research foundation, featuring problem-based learning and collaborative learning approaches, the course “Practical Chinese for engineering students” aims to help students, through lectures, tutorials, role plays and cases-based workshops, develop the ability to use the Chinese language effectively in the workplace.

The purpose of this study was to investigate the possible use of online PBL and how this may support students’ technical communication and writing skills in undergraduate Engineering. We developed the following hypothesis: the online PBL learning method can provide students with a stronger sense of classroom belonging and positively impact students’ psychological health during the COVID-19 quarantine, so that students can focus on learning and thus improve their language ability. To fulfill this aim, the following specific research questions were formulated:

RQ1: How does online PBL influence the test scores of students’ language ability?
RQ2: How does online PBL influence students’ sense of classroom belonging?
RQ3: How does online PBL help students create feelings of connection and “togetherness”?

## 4. Research Design

### 4.1. Participants and Procedure

A total of 44 participants, all members of the class of 2019 from the school of engineering at The University of Hong Kong (HKU), took part in this study. The 44 respondents were randomly divided into two groups—the experimental group (C1) and the control group (C2)—with 22 students in each group (as can be seen in Table 1). The experimental group included 10 males and 12 females, while the control group included 11 males and 11 females. Therefore, the gender ratios of the two groups were similar. We implemented online PBL learning for the experimental group, and traditional online teaching for the control group. The course content, test questions, and tutor were the same for the two groups. All 44 students come from the same class. The students were assigned to each group based solely on their student number (UID), and they were not informed that they would be part of an experiment. Ethics approval was granted by the department. Considering that students are unfamiliar with the term PBL, the tutor uses “group discussion” or “tutorial” instead. The experiment lasted one semester, from February 2020 to April 2020, including 12 teaching weeks in total.

During the quarantine, the university courses were all delivered via Zoom, a web conferencing application incorporating a range of functions such as chat, video interaction and interactive whiteboards. Conventional methods were used in the control group, where the instructor lectured most of the time, gave some pre-reading assignments and managed small class discussions in class. Information related to the course was posted via the Moodle learning system. The lecturer and the tutor were jointly responsible for addressing students’ questions regarding the material delivered via the weekly lessons. Basically, in the control group, a traditional face-to-face teaching method was reproduced in an online form.

The teaching steps for the experimental group were as follows: (1) Every team was assigned four PBL projects, the completion of which required joint discussions and cooperation—every PBL project included several subsidiary topics; (2) Students were asked to share their knowledge and progress via the Moodle forum, and WhatsApp groups were established for after-class discussions; (3) Every team was required to finish four project reports and upload them to the Moodle learning management system at the end of the term; (4) The tutor assessed students’ performance on the PBL projects. As can be seen in Table 2, there were a total of four modules, which focused on different aspects of language practice and writing.

### 4.2. Instrumentations

The study was conducted as a mixed-methods study. We used a convergent parallel design and conducted the quantitative and qualitative elements in the same phase of the research process. Both the quantitative and qualitative parts of the study were equal. We analyzed them independently and presented data together. This approach allows us to evaluate the perspective at different levels of reality and explore the reasons behind the observed effect [22]. Concretely, the quantitative part of the study answers questions about if and how online PBL influences students’ learning outcomes and the qualitative part of the study provided us with answers to why this happens. Three data sources were used to assess the effectiveness of the online PBL approach: (a) achievement test data, (b) a classroom community scale, (c) a focus group discussion (FGD). 

#### 4.2.1. Pre- and Post-Standardized Scoring Tests

A standardized scoring test was the primary method of our data collection. In order to get a picture of PBL’s influence on students’ learning outcome, we designed a pre-pro test (a multiple-choice knowledge and comprehension test) to assess student learning. The pre-test and post-test questions were prepared by the course coordinator and posted on the Moodle learning system. The test consisted of 25 items (10 knowledge levels such as traditional Chinese characters, simplified Chinese characters, modern Chinese grammar, Cantonese phonetics, and 15 comprehension level items, such as the different uses of vocabulary and expressions between the Cantonese dialect and Mandarin in the engineering workplace context). All the questions come from a valid and standard question pool of the Chinese Language Center in HKU which has been used to test students’ command of Chinese for the STEM profession for many years.

#### 4.2.2. Classroom Community Scale

We used a “Classroom Community Scale” (CCS) designed by Rovai to measure the sense of classroom belonging in the online learning environment [23]. Rovai defined the sense of classroom community as the feeling that members have a belonging; that members matter to one another in the group; and that members’ needs will be met through the commitment of being together in the learning process [23]. The CCS was found to be an effective tool to measure classroom belonging, particularly in university courses taught asynchronously via the internet. The CCS has two dimensions—sense of connectedness and sense of class learning—with a total of 20 items on 5-level Likert scales. Connectedness (odd-numbered items) represents the feelings of community among students regarding their connectedness, cohesion, spirit, trust, and interdependence. Learning (even-numbered items) represents the community members’ feelings about their interactions with each other as they pursue the construction of understanding and the degree to which members share values and beliefs concerning the extent to which their educational goals and expectations are being satisfied.

The CCS was first developed in 2002 by Rovai and, in 2004, Rovai, Wighting, and Lucking refined and further validated the scale [24]. During this process, the scale was evaluated by a panel of four experts at face value for validity. The experts found that the scale posed a high level of content validity. The CCS was also evaluated for concurrent validity by determining its ability to vary indirectly when measuring opposite concepts. During the study, a Cronbach’s coefficient α was conducted to evaluate reliability. The Cronbach’s coefficient α reliability for the two groups was 0.84 and 0.83, respectively. In addition, the internal consistency coefficients for social learning and community learning for the subgroups were 85 and 82, respectively. In order to evaluate stability, a Pearson’s r correlation coefficient utilizing a pre/posttest model was given over two-week intervals. The stability for each interval was 0.91 [24,25]. CCS has already been used in many studies related to online learning in the field of higher education [25,26,27,28]. Wiest completed a comparative study of classroom community in a fully blended online social work course versus a traditional social work course at a nonprofit Catholic institution in Central Florida [25]. Deng conducted research using the CCS to assess the influence of online PBL on students’ classroom belonging to a physics pedagogy course in China [28]. All results support the instrument as valid, reliable and stable, which made it an optimal tool for our study. As the working language of this Hong Kong University is English, the items of the scale need not be translated. In a word, the Classroom Community Scale responds well to our needs.

#### 4.2.3. Focus Group Discussion

For the qualitative part, to find out what students think about the PBL learning method, eight participants (four males and four females) from the experimental group were interviewed via Zoom. A focus group approach was selected over individual interviews due to the interactive quality of this method, thus offering students the opportunity to discuss and express feelings. This method has the advantage that group interaction can generate more diverse and in-depth perceptions as well as individual experiences and opinions. It brings together a small group of people, usually to answer a series of questions and report on the responses. The ideal number of focus group interview participants is between six and twelve [29].

Students were also encouraged to use whatever colloquial diction and words came naturally to them. The themes for the interview guide were developed from the research objectives and from the literature review of the subject area. They were then used as a prompt throughout the interviews. The interview session was video-recorded with the participants’ approval. Data were transcribed into NVivo 11 software to present visualizations for a deeper understanding of emerging themes.

Following the interviews, the video recordings were transcribed verbatim by the authors, and the transcribed raw data were then analyzed following the process of thematic analysis, which is an inductive approach to data analysis. The six phases of thematic analysis as described by Braun and Clarke were followed [30]. The three authors read and reviewed all of the interview data independently and thoroughly to develop an emergent set of phenomena. In particular, in order to scrutinize and verify meaningful data, line-by-line coding was used to engage the qualitative data in depth. Then conceptually similar codes were grouped together. Additionally, the authors refined, aligned, and connected codes to develop core themes. Finally, the authors discussed and identified a higher level of conceptual themes to generate core and central concepts and phenomena. Discussion continued amongst the authors until encompassing or major themes were adequately obtained. As a result of the data analysis process, the authors constructed three themes related to the interviewees’ experiences of teaching online during the pandemic.

## 5. Results

### 5.1. Online PBL Influence on the Test Scores of Students

Quantitative survey data were analyzed descriptively using SPSS 22. The pre-test can be used as a tool to control variables. The average scores of the experimental group and the control group were 66.680 and 64.180 respectively. The students were randomly assigned to each group based on their student number (UID). Based on the two-sample t-test, the average scores of the pretest of the two groups are not statistically different (see Table 3). Therefore, the value in the experimental class, and the pre-test value in the control class has the same ability based on the test difference of the two average pre-test scores. The experimental and control groups in this study had a comparable starting point.

The means and standard deviations for the pre–post test for both C1 and C2 groups are given in Table 4. It is clear that the gains in the experimental group’s scores (+8.27) were higher than those of conventional students (+2.775). Paired samples t-tests confirmed the differences between the pre-test and post-test scores for the experimental and control groups. This indicates that the use of the PBL learning model has a better outcome than the conventional learning models.

### 5.2. Online PBL Influence on Students’ Sense of Classroom Belonging

The validated Classroom Community Scale examined students’ self-reported sense of connectedness and learning. The questionnaire was conducted among experimental and control group respondents on 15 April 2020, one month before the final-term examination. The means and standard deviations for the Classroom Community Scale for both C1 and C2 groups are given in Table 4 and Table 5. As the two tables show, we found significant differences between the experimental and control groups in sense of both connectedness and learning. This suggests that PBL can increase the sense of connectedness and learning among students in the experimental group and enrich their socializing experience.

The least favorable choice is always assigned a value of 1 and the most favorable choice is assigned a value of 5. Connectedness and learning subscale scores can each range from 10 to 50, with higher scores reflecting a stronger sense of classroom belonging. Table 6 shows descriptive statistics of the Classroom Community Scale.

Based on these results, the answer to research question two is that the online PBL learning method: (1) motivated the participants to learn; (2) enhanced the participants’ sense of classroom community; and (3) involved the participants in the learning tasks. It is clear that the experimental students’ scale scores were higher than those of the control group students on most items. Item 2, “I feel connected to others in this course,” had the highest mean score (M = 4.05). This indicates that, by producing the same sense of security and trust as it does in an in-person class, PBL enabled the experimental group to have a more satisfactory and closer-to-in-person-class experience than the control group. This answer aligns with the qualitative data presented in the answer to research question three.

### 5.3. Online PBL Help Students Create Feelings of Connection and Togetherness

Answering our third research question, three main themes and eight sub-themes emerged, including: (a) general views on distance learning; (b) views on PBL group work; and (c) learning effects of this course (as can be seen in Table 7). Each theme is discussed separately below.

**Theme** **1.**  *General views on distance learning*.

The students discussed their general views on distance learning during quarantine. Comments related to this main theme were grouped into three subthemes: lack of social interaction, panic and fear, and missing campus.

i. Lack of social interaction. The students felt it was difficult to concentrate and maintain motivation for long periods. They attributed this situation to staying home alone in front of a small screen rather than with others in a physical learning space for a specific time. Two students said that “I sit at home alone all day. Not socializing has become a big problem. It is really hard to concentrate and stay focused” (S3) and “Every day, I dress in nightwear, sit in front of my laptop, and listen for one and a half hours to an online lecture. I have tried many ways to keep myself focused, but all failed” (S5).
ii. Panic and fear. Students found online lessons isolating when they were not interacting with their classmates or the teacher. More seriously, as the anti-government protests and the spread of COVID-19 continued, students felt more worried and scared. Two students mentioned “Like many other Hong Kongers, I have been overwhelmed by a sense of helplessness and anxiety during the past four months” (S1) and “As the protests turn to chaos and violence, you can’t help feeling distracted at home when learning via online instruction” (S8).
iii. Missing campus. The students missed the variations in and interactions from on-campus learning activities. For instance, students expressed that they missed the variation in having to get up in the morning to go to campus. One student mentioned “When I saw masked protesters setting fire to the entrance of our university on the news, I felt very disturbed and mostly scared. I miss campus so much. I’m not sure when I’ll be back in class” (S4).

**Theme** **2.**  *Views on PBL group work*.

Two subthemes emerged from students’ descriptions of group behavior and experiences, which were interaction and productivity.

i. Interaction. As the students’ learning activities centered around their PBL project, they met frequently online to engage with each other. One student said, “It would be nice to talk to group members during quarantine because I know we’re going through a similar experience together at this moment.” (S8) Students worked in small teams, collaborating, communicating, and sharing information. We also found that students followed one another’s progress on the course forum. The students claimed that they “express their differing opinions freely in groups” (S7), and in return, it led to more interaction. Such connections between students in online education could positively promote their learning. One student expressed that “It’s really hard to concentrate during distance classes. After all, language learning, such as modern grammar and rhetoric, is a bit boring. The form of collaborative learning is a good solution to this problem. After a long time of isolation at home, I really enjoy communicating with my classmates” (S5). There is no doubt that cooperative learning in an online environment has a positive effect on students’ attitudes and emotions.
ii. Productivity. Students stressed the responsibilities of group members when they talked about teamwork. They stated that the success of team work largely depends on the individual work of team members. Self-control becomes an integral part of the process. The student reported that when some group members did not want to continue with teamwork, other members encouraged and helped them complete their parts of the task. One student shared this story: “One day, I saw an COVID-19 quarantine announcement issued in our community. I was in a state of panic and did nothing the whole day. Then, one of my group members asked me about the progress of my assignment on Moodle. This gave me a sense of connection that made me feel as if I had returned to campus and was temporarily free from my limited, fear-filled life. I then started concentrating on courses. (S2) The PBL group members were always accountable toward one another on an ongoing basis to produce results (i.e., the practical solution to the case, the writing assignment, and the presentation). This accountability meant that the students committed to keeping up with online lectures and exercises during the lockdown, as they needed knowledge for their shared PBL project. A student mentioned that “in order to submit the final group work project, we must ensure that each team member is working hard on the task. If there is slow progress, we will @ him or her in the WhatsApp group” (S8). The students mentioned that online group work seemed to generate a “work hard” dynamic for them.

**Theme** **3.**  *Learning effects of this course*.

Three subthemes were identified: enhancing language proficiency, surprised and satisfied, and workload.

i. Enhancing language proficiency. The students reported that they have become more confident about their ability to use the Chinese language effectively upon entering an engineering workplace after this course. Some students described feeling confident communicating after this course with comments such as “the cases-based group workshop is quite interesting. I learnt how to be an effective technical negotiator” (S1) and “I have a solid grasp of all the technical terms in simplified written forms required in the syllabus” (S2). One student said, “I really think this course is very interesting and interactive, not just sitting for an exam. We really have to act like an engineer to give a presentation in the simulation work circumstance” (S5).
ii. Surprised and satisfied. Some students expressed surprise and satisfaction with their experience with PBL online. Comments included, “I was quite surprised it worked so well” (S4), and “I’d say there are indeed benefits” (S7). One student said, “Since the very beginning, we didn’t know what to do, we were confused…but eventually this course was not as terrible as I had imagined. It was interesting to summarize the key points of the theoretical part by sharing different opinions during teamwork” (S6).
iii. Workload. Even though the students found their online group meetings positive and productive, some students complained about the workload of the course. For example, “The only problem is the workload. Looking at the computer screen for a long time is very harmful to my eyesight. After all, I have seven compulsory courses this semester. Seminar assignments for this course can be cut in half” (S1). As screen time increases, students are more likely to encounter the problem of eye strain and visual fatigue. Thus, group assignments should be adequately planned and monitored to avoid overloading the students.

In summary, the qualitative findings derived from the content analysis of the focus group discussions support the quantitative findings. All participants thought that the tutorial (PBL) learning approach used in this course was better than the traditional way to learn and increased their level of proficiency in the specialized usage of Chinese in the field of engineering and that it led to more participation. No respondents gave an explicitly negative evaluation. To this extent, the online PBL learning method could help increase students’ devotion to learning.

## 6. Discussion

PBL is considered an active learning strategy compared to other approaches to learning. Its use in a teaching context allows the student to move beyond a passive role, to be exposed to a problem and to develop a sense of self-direction in search of the knowledge required to solve it [6]. As mentioned in the literature review, one of the main virtues of PBL is that it displays a significant advantage over traditional methods in how students’ communication skills are improved. The general ability for social interaction is also positively affected. These are two central factors in language learning [31]. By building a language course around outcome-based assignments that require students to act, interact, and communicate, it is hopefully possible to mimic some of the aspects of learning a language “on site,” that is, in a workplace where this technical language is actually used. Learning practical language in such an environment is obviously much more effective than just being fed specialized terms and expressions without context. Thus, PBL group projects can promote important language and social skills for students to solve uncertain, complex, or open-ended workplace problems. Therefore, engineering students could be exposed to curriculum activities to become proficient communicators in professional settings.

The result of this study generally coincides with the findings of Rovai [23], showing that a sense of classroom belonging can be acquired through team building and communication among teammates. Additionally, the feeling of being an integral part of their group also motivated the students to learn in a way that the prospect of a final examination rarely manages to do. In previous research, rapport building was identified as more difficult online than in person due to a diminished sense of bonding [17]. Although students can communicate through video, text, and audio in an online environment, there is a lack of directness that comes from face-to-face communication and the richness of emotions. PBL has the potential to emphasize the affective traits of what is learned and the learning experience itself, while promoting the willingness and desire to learn. As students support and encourage one another, their interpersonal relationships become well-developed, and the awkwardness among students disappears naturally. They can then express their views on problems with confidence. Thus, through the PBL method, students can create a sense of community and sustain strong ties through online courses.

Williamson and Gregory found that students in PBL find cooperative efforts taxing; they fear free-loaders and would rather rely on their own abilities [32]. However, in a long-term network learning environment, students are not reluctant to engage in group work. The students state that their interactions with each other and their intra-group support for the sake of group success made it necessary for every individual to focus on their learning.

Engineers often work in team settings. Beyond the skills that are necessary to excel as technical contributors, engineers need negotiation skills. A structured PBL design is needed for engineering faculty members and universities to develop students’ professional knowledge and transferable skills. In this study, it was observed that as students discussed how to solve the problem in small groups of four or five, they began to gain better practical language skills through the brainstorming of ideas. The PBL assignment provided a platform for students to express their ideas and to share responsibility in managing problem situations. Different views and expressions on a case were observed, leading students to ask new questions. This helped to facilitate greater interpersonal communication and develop the group dynamic.

In summary, the online PBL learning method managed to provide a social structure that gave students the opportunity to: (1) work closely with their peers in a group; (2) apply knowledge and expressions gained from lectures to practical problems; and (3) collaborate to understand the problem’s complexity and potential solution in a real-world setting.

## 7. Conclusions and Limitations

Innovative educational adaptations have been essential during the COVID-19 pandemic. In this study, we implemented online PBL learning for the experimental group, C1, and traditional online learning for the control group, C2. Quantitative data were collected via an achievement test and a scale to sense the classroom community. In the achievement test, while students’ post-test scores were higher than their pre-test scores in both the experimental and control groups, the difference in the experimental group’s post-test scores was more significant. This suggests that PBL improved the test scores of students receiving online education more effectively than the traditional approach. The classroom community scale revealed that PBL led students in the experimental group to engage more actively in online learning. Students’ scores in the sense of connectedness dimension were obviously higher in the experimental group. By and large, this study’s experimental data found that the PBL learning method was effective in promoting students’ deep active learning and sense of community in the online environment.

The qualitative analysis complemented the quantitative analysis and helped us further explore and explain the quantitative data and to gain a deeper understanding of the significant findings by considering and analyzing students’ feelings. In the focus group discussion, the students reported that the PBL learning approach was indeed crucial for student engagement. Their digital connection to their PBL group “saved them each day” during the quarantine since it meant they were not alone with their tasks or their social and emotional needs. In that sense, our mixed methods findings showed complementarity in terms of elaborating on and enhancing results, as well as revealing a more complex picture [33].

Our findings suggest that PBL improves technical language learning by shifting away from a lecture-based pedagogy of facts and definitions toward a more student-centered approach aimed not only at increasing students’ knowledge of cutting-edge technology but also professional communication and writing skills. In this sense, while PBL is applicable in a variety of fields, it is particularly suitable in practical language classes, especially for STEM purposes.

The most significant limitation of this study is the small sample size used. In particular, this course for language skills development was conducted during the unusual period of the COVID-19 pandemic. Students in this study come from the same class and have already developed and maintained good relationships with others. The replication of this research might reveal different results depending on different students and social backgrounds. We hope that others can learn from our work and use it to prepare for suspension of face-to-face classes, whether caused by the COVID-19 pandemic or other health emergencies.

## Figures and Tables

**Table 1:** Characteristics of participant student groups.

Group	No. of Students	Academic Year	Group Formation
C1	22	2020 Spring Semester	Randomly allocated
C2	22		

**Table 2:** The tutorial designed for the experimental group.

Phase	Description
Formation of tutorial groups 1st class	Students were divided into five teams of four or five people each.
1st tutorial 3rd class	Every team was asked to summarize the characteristic styles and formats of practical Chinese writings in the workplace context in Hong Kong in the field of engineering, and then develop a report together.
2nd tutorial 6th class	Every team was asked to develop a report to list several useful engineering terminologies in their traditional and simplified Chinese forms.
3rd tutorial 9th class	Students would be given a task to “improvise” a scenario related to communication or professionalism in a given context followed by self-reflection and feedback from other students in the group.
4th tutorial 12th class	Every team was asked to finish a report writing assignment (draft + final) together to heighten the students’ linguistic sensitivity. Explicit writing instructions were given by the tutor.

**Table 3:** Two sample *t*-Test of the Pre-test Results for C1 and C2.

Type	Group	N	Mean ± SD	df	T Stat	*p* (Two-Tail)
C1	Pre-test	22	66.680 ± 211.465	42	0.604	*p* = 0.549
C2	Pre-test	22	64.180 ± 165.489			

**Table 4:** Paired samples *t*-Test of the Pre- and Post-test Results for C1 and C2.

Type	Group	N	Mean ± SD	df	*t*	*p*
C1	Pre-test	22	66.680 ± 14.542	21	−2.131	*p* = 0.045
	Post-test	22	74.955 ± 12.511			
C2	Pre-test	22	64.180 ± 12.864	21	−0.751	*p* = 0.461
	Post-test	22	66.955 ± 13.668			

**Table 5:** Impact of PBL on the Sense of Connectedness and Class Learning.

Constructs	Group	Mean ± SD	Max.	Min.	*t*	*p*
Connectedness	C1	36.64 ± 5.55	43	23	5.689	*p* < 0.001
	C2	26.73 ± 5.99	40	20		
Learning	C1	33.91 ± 4.26	40	24	3.450	*p* = 0.001
	C2	28.68 ± 5.69	40	20		

**Table 6:** Descriptive Statistics of the Classroom Community Scale items.

	Mean ± SD		*t*	*p*
	C1 (n = 22)	C2 (n = 22)		
1. I feel that students in this course care about each other	3.82 ± 0.85	2.36 ± 0.95	5.333	*p* < 0.001
3. I feel connected to others in this course	4.05 ± 0.84	2.41 ± 0.91	6.191	*p* < 0.001
5. I do not feel a spirit of community	3.59 ± 0.91	2.77 ± 0.75	3.255	*p* = 0.002 **
7. I feel that this course is like a family	3.27 ± 0.83	2.50 ± 0.67	3.400	*p* = 0.001 **
9. I feel isolated in this course	3.86 ± 0.83	2.86 ± 0.83	3.979	*p* < 0.001
11. I trust others in this course	3.50 ± 1.06	2.91 ± 0.68	2.200	*p* = 0.034 *
13. I feel that I can rely on others in this course	3.59 ± 0.73	2.73 ± 0.83	3.663	*p* = 0.001 **
15. I feel that members of this course depend on me	3.41 ± 0.91	2.73 ± 0.88	2.525	*p* = 0.015 *
17. I feel uncertain about others in this course	3.82 ± 1.01	2.77 ± 0.87	3.687	*p* = 0.001 **
19. I feel confident that others will support me	3.73 ± 0.83	2.68 ± 0.72	4.482	*p* < 0.001
2. I feel that I am encouraged to ask questions	3.55 ± 0.80	2.77 ± 0.81	3.178	*p* = 0.003 **
4. I feel that it is hard to get help when I have a question	3.41 ± 0.91	2.82 ± 0.73	2.375	*p* = 0.022 *
6. I feel that I receive timely feedback	3.18 ± 0.66	2.82 ± 0.85	1.578	*p* = 0.122
8. I feel reluctant to speak openly	3.18 ± 0.91	2.86 ± 0.77	1.251	*p* = 0.218
10. I feel uneasy exposing gaps in my understanding	3.23 ± 0.69	2.86 ± 0.89	1.520	*p* = 0.136
12. I feel that this course results in only modest learning	3.27 ± 0.83	2.82 ± 0.80	1.858	*p* = 0.070
14. I feel that other students do not help me learn.	3.45 ± 0.67	2.86 ± 0.64	2.990	*p* = 0.005 **
16. I feel that I am given ample opportunities to learn	3.50 ± 0.86	3.00 ± 0.69	2.128	*p* = 0.039 *
18. I feel that my educational needs are not being met	3.50 ± 0.91	2.91 ± 0.75	2.346	*p* = 0.024 *
20. I feel that this course does not promote a desire to learn	3.64 ± 0.73	2.95 ± 0.72	3.121	*p* = 0.003 **

* *p* < 0.05, ** *p* < 0.01.

**Table 7:** Thematic analysis of focus group discussions.

Themes	Sub-Themes/Categories	Exemplar Quotes
General views on distance learning	Lack of social interaction	“Not socializing has become a big problem. It is really hard to concentrate and stay focused.”
	Panic and fear	“I was in a panic and did nothing the whole day.” “I have been overwhelmed by a sense of helplessness and anxiety during the past four months.” “As the protests turn to chaos and violence, you can’t help feeling distracted at home when learning via online instruction.”
	Missing campus	“I miss campus so much. I’m not sure when I’ll be back in class.”
Views on PBL group work	Interaction	“I think the most important thing in this course is communication, and it is more of an interactive session.” “Group discussion meant that we were not alone.”
	Productivity	“If there is slow progress, we will @ him or her in the WhatsApp group.”
Learning effects of this course	Enhancing language proficiency	“I really think this course is very interesting and interactive, not just sitting for an exam. We really have to act like an engineer to give a presentation in the simulation work circumstance.”
	Surprise and satisfaction	“I was quite surprised it worked so well.” “I’d say there are benefits.” “Group work makes it definitely much easier to catch up.”
	Workload	“The only problem is the workload (brought on by group work). Seminar assignments for this course can be cut in half.”

## Data Availability

Not applicable.
